# Alcohol Inhibits Organic Dust-induced ICAM-1 Expression on Bronchial Epithelial Cells

**DOI:** 10.3390/safety3010005

**Published:** 2017-01-07

**Authors:** Todd A. Wyatt, Kerry Canady, Art J. Heires, Jill A. Poole, Kristina L. Bailey, Tara M. Nordgren, Debra J. Romberger

**Affiliations:** 1 Pulmonary, Critical Care, Sleep & Allergy Division of the Department of Internal Medicine, University of Nebraska Medical Center, Omaha, NE, 68198; USA; 2 Department of Environmental, Agricultural, & Occupational Health, University of Nebraska Medical Center, Omaha, NE, 68198-5910; 3 Resereach Service, VA Omaha-Western Iowa Health Care System, Omaha, NE, 68105

**Keywords:** bronchial epithelial cells, organic dust, ICAM-1, alcohol, inflammatory lung dieseas

## Abstract

Aims: Exposure to dusts/bioaerosols in concentrated animal feeding operations (CAFOs) results in inflammatory lung diseases in workers. Hog CAFOs dust extract (HDE) increases expression of intercellular adhesion molecule-1 (ICAM-1), neutrophil adhesion, and TNFα release in bronchial epithelial cells. Alcohol consumption is increasingly recognized to impair lung immunity. We hypothesized that alcohol impairs HDE-induced TNFα, ICAM-1 expression and neutrophil adhesion by directly inhibiting TNFα converting enzyme (TACE) activity.

**Methods:**

Bronchial epithelial cells (BEAS-2B) and primary human bronchial epithelial cells were pretreated with ethanol (EtOH) or TACE inhibitor. ICAM-1 surface expression, TNFα release, and TACE activity were analyzed following HDE stimulation. The effect of alcohol and TACE inhibition on HDE-regulated epithelial cell/neutrophil adhesion interactions was investigated. Finally, utilizing an established animal model, C57BL/6 mice were fed *ad libitum* ethanol (20%) in drinking water for 8 wk followed by daily intranasal inhalation of HDE or saline during the final two weeks. Mice were sacrificed and lung sections immunostained for ICAM-1.

**Results:**

Pretreatment with alcohol or TACE inhibitor significantly decreased HDE-induced ICAM-1 expression and TNFα release. HDE augmented neutrophil adhesion to epithelial cells, which was decreased with alcohol (32% decrease) or TACE inhibitor (55% decrease) pretreatment. TACE activity increased following HDE exposure, but TACE activity was inhibited following alcohol pretreatment. Alcohol-fed mice demonstrated decreased HDE-induced airway epithelium ICAM-1 expression.

**Conclusions:**

Alcohol diminishes HDE-induced ICAM-1 expression, TNFα release, and neutrophil adhesion via inhibition of TACE activity. These results suggest that alcohol may be an important modulator of lung innate immune responses following CAFO exposure.

## 1. Introduction

Agricultural workers experience significant occupational health risks, not the least of which are chronic lung diseases associated with the agricultural production environment. Large-scale meat production involves the use of concentrated animal feeding operations (CAFOs) resulting in the concentration of animal-generated gasses (such as ammonia, hydrogen sulfide, and carbon dioxide) and dusts (consisting of livestock-generated dander, bacterial, and fecal components) [1]. CAFO workers consistently demonstrate poor respirator compliance due to the physical demands of the work making any airflow reduction fairly intolerable [2]. Because of increased concentration and duration of exposure, CAFO shift workers are at elevated risk of dust inhalation. Inhalation of these dusts has been associated with enhanced chronic inflammatory lung diseases including chronic bronchitis, occupational asthma, hypersensitivity pneumonitis, and organic dust toxic syndrome [3].

An increasingly recognized important modulator of lung responses to injury and disease is alcohol. Alcohol alters numerous mechanisms of innate and adaptive immunity in the lung [4]. A significant association has been demonstrated between alcohol consumption and work-related injuries among farmers [5]. In a Colorado study of 872 farmers, person-days of alcohol drinking was reported as 48% had no drinks (abstinence), 41% had 1–2 drinks per day (moderate drinkers) and 11% had 3 or more drinks per day (heavy drinkers) [6]. These data show that the majority of farmers are consuming alcohol (52.1%). In an Australian study, farmers were found to be much more likely to drink excessively than people living in urban areas [7]. Such findings are not surprising in that farming is considered to be a high-stress profession associated with increased rates of depression and suicide [8]. As an etiology of alcohol consumption and alcohol use disorders, stress is an established health risk leading to disease [9, 10]. Based upon these reports, a consideration for the role of alcohol consumption in the context of agricultural organic dust inhalation-induced injury is necessary.

Lung innate protection against chronic injury can be negatively impacted in several ways by agricultural organic dusts. Mucociliary clearance is diminished in response to inhaled organic dust as swine barn dusts have been shown to slow cilia beating via the activation of protein kinase C epsilon [11]. Wound repair can be detrimentally impacted as swine barn dust exposure slows airway epithelial cell migration into a wound [12]. In previous studies we observed that extracts of dust collected at hog CAFOs (HDE) elicit an inflammatory response in bronchial epithelial cells characterized by increased expression of intercellular adhesion molecule-1 (ICAM-1), neutrophil adhesion to epithelial cell monolayers, and the production of pro-inflammatory cytokines, including IL-6 and IL-8 [13]. The HDE-stimulated release of IL-6 and IL-8 requires a sequential pathway whereby dust-mediated release of TNFα activates PKCε in an autocrine manner to stimulate interleukin release [14]. Recently, we have demonstrated that cytokine release in response to HDE can be blunted by cAMP-dependent protein kinase inhibition of ADAM-17, the sheddase responsible for TNFα release [15]. Furthermore, we have shown that alcohol-stimulated increases in cAMP can lead to a blunted PKC-mediated cytokine response in HDE-exposed airway epithelium [16]. These observations provide the mechanistic basis for the dysfunctional lung inflammatory response observed in alcohol-fed mice exposed to swine barn dust [17]. Because alcohol can impair the release of cytokines instrumental in critical inflammatory reactions, we hypothesized that alcohol would impair HDE-induced ICAM-1 expression and neutrophil adhesion by directly inhibiting TNFα converting enzyme (TACE) activity in bronchial epithelial cells both in culture and in *in vivo* exposure models.

## 2. Results

### 2.1 Alcohol dose-dependently alters HDE-stimulated PKC activity and cytokine release

**Figure 1:**
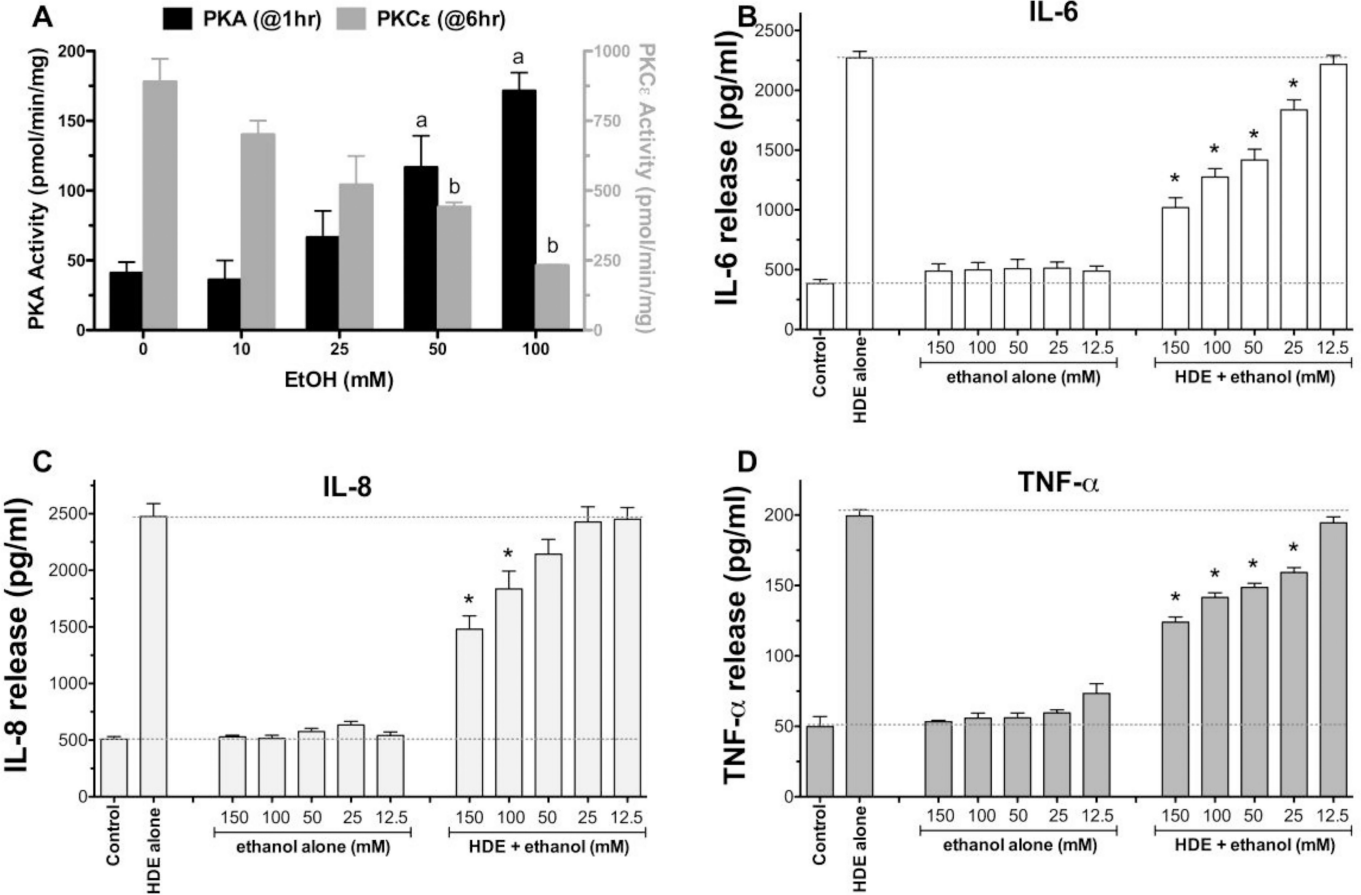
Ethanol dose-dependently activates PKA and inhibits HDE-induced cytokine release from BEAS-2B cells BEAS-2B were pretreated with various concentrations of EtOH (12.5 to 150 mM) for 1 h before being exposed to 5% HDE (± EtOH) for an additional 24 h. EtOH dose-dependently increases PKA activity at 1 hr (A; black bars) and decreases HDE-stimulated PKC epsilon activity at 6 hr (A; gray bars). Inflammatory cytokines TNFα (B); IL-6 (C); IL-8 (D) measured in culture supernates by ELISA. ^a^p<0.05 vs 0 mM EtOH for PKA; ^b^p<0.05 vs 0 mM EtOH for PKCε; *p<0.05 vs HDE alone for n=3 or 4 independent experiments (ANOVA, Tukey’s post test).

Bronchial epithelial cells (BEAS-2B) were pretreated for 1 hr with or without 0–150 mM ethanol (EtOH), followed by exposure to 5% HDE or control media for 1, 6, and 24 hr. Consistent with previous findings, alcohol dose-dependently and rapidly (1 hr) activated PKA [24], but inhibited HDE-stimulated PKCε [17] (Fig 1A). Similarly, alcohol pretreatment dose-dependently decreased HDE-stimulated TNFα, IL-6 and IL-8 (Fig 1B–D). No cell toxicity was detected under any concentration of alcohol used. Because the 100 mM EtOH concentration was consistently effective at inhibiting all three HDE-stimulated cytokines with no observed cytotoxicity, all subsequent experiments used that optimized concentration.

### 2.2 Alcohol exposure decreases HDE-mediated ICAM-1 expression

**Figure 2:**
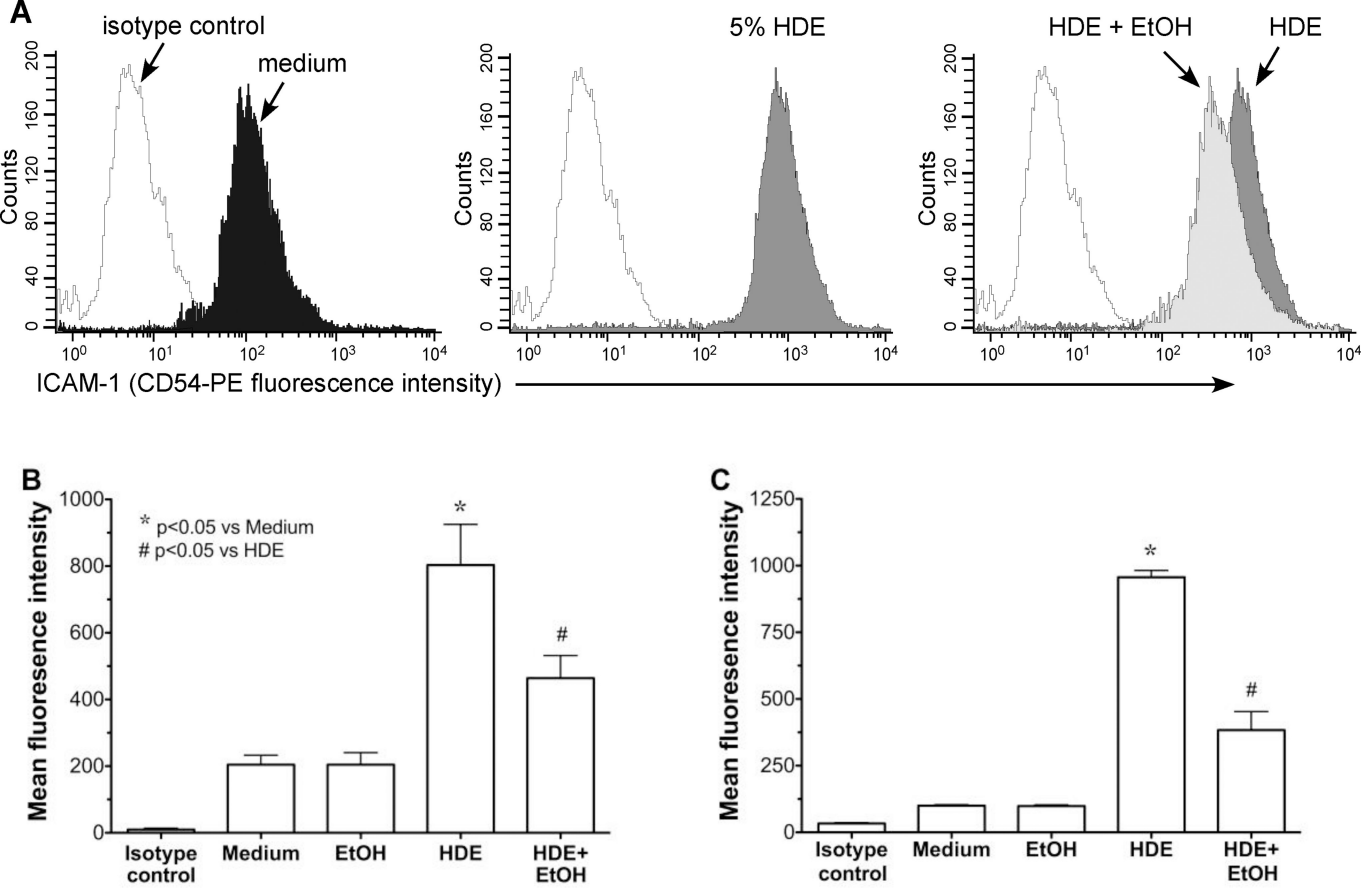
Alcohol pretreatment down regulates HDE-mediated ICAM-1 expression in human bronchial epithelial cells BEAS-2B (B) and HBEC (C) were treated with control medium or 5% HDE +/− 100 mM ethanol (EtOH) for 24 hr and FACS analysis was performed. (A) Representative histogram showing significant rightward shift following HDE exposure (dark gray histogram), where open histograms represent isotype control antibody. Panel on far right depicts HDE treatment (dark gray) compared to EtOH+HDE treatment (light gray). Bar graphs depict means with standard error bars from N=6 (B) and N=3 (C) independent experiments. *p<0.05 vs. control medium, ^#^p<0.05 vs. HDE.

To examine the effects of alcohol on HDE-induced ICAM-1 expression, both cultured BEAS-2B cells and primary HBECs were pretreated for 1 hr with or without 100 mM ethanol, followed by exposure to 5% HDE or control media for 24 hr. Cells were fixed and analyzed by FACS for ICAM-1-specific immunostaining.HDE induced the upregulation of ICAM-1 surface expression in BEAS-2B as determined by the rightward histogram shift in fluorescence intensity counts for each individual experiment (Fig 2A). Alcohol pretreatment significantly (p<0.05) inhibited the HDE-induced shift in ICAM-1 expression as determined by mean fluorescence intensity (MFI; Fig 2B). As a control, alcohol itself had no effect on the baseline ICAM-1 expression. Consistent with these findings, alcohol pretreatment also reduced HDE-stimulated surface expression of ICAM-1 in primary HBECs (Fig 2C). These data demonstrate that alcohol exposure reduces HDE-stimulated ICAM-1 expression in airway epithelial cells.

### 2.3 Alcohol decreases HDE-mediated neutrophil adhesion

**Figure 3:**
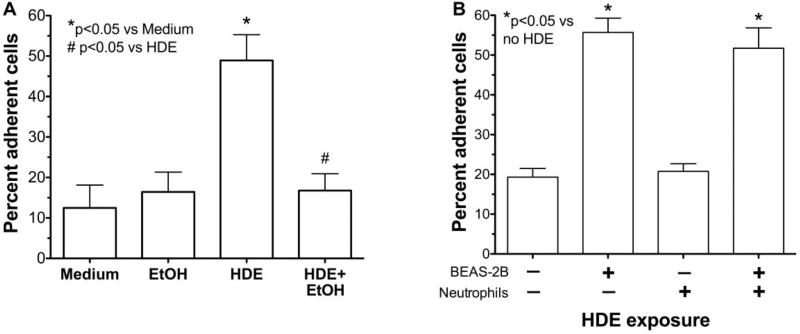
Alcohol inhibits HDE-induced peripheral blood neutrophil adhesion to epithelial cells (A) BEAS-2B cells were pretreated with 100 mM ethanol (EtOH) prior to exposure to 5% HDE or control medium for 24 hr. Neutrophils were then allowed to adhere to stimulated BEAS-2B for 30 min. HDE causes a significant increase in PMN adhesion compared to medium alone. Pretreatment with EtOH causes a significant decrease in HDE induced adhesion. (B) Neutrophils were treated directly with 5% HDE and then incubated with BEAS-2B. Treatment of neutrophils with HDE does not stimulate adhesion to untreated BEAS-2B. Bar graphs represent means with standard error bars of 4 independent experiments, *p<0.05 vs. control medium, ^#^p<0.05 vs. HDE.

Neutrophil adhesion to the airway epithelium is mediated by ICAM-1and HDE-induced ICAM-1 expression is reduced in the presence of ethanol (Figure 2). Thus, we investigated whether HDE-stimulated neutrophil adhesion is also attenuated by ethanol pretreatment. Compared to medium treatment alone, a 24 hr treatment with 5% HDE stimulates greater than a 4-fold increase (p<0.05) in neutrophil adhesion to BEAS-2B cells (Fig 3A).However, a 1 hr pretreatment with 100 mM ethanol significantly (p<0.05)abrogated HDE-stimulated increases in neutrophil adhesion to BEAS-2B. Importantly, pre-incubation of BEAS-2B with ethanol alone produced no change in neutrophil adhesion compared to control medium-treated BEAS-2B, suggesting that alcohol does not directly alter epithelial-neutrophil adhesion due to artifact. In addition, neutrophils were not exposed to HDE at any time for these assays. As a control, we exposed neutrophils directly to HDE to determine if the dust could activate neutrophils, leading to an increase in adhesion. We observed an increase in adhesion only when epithelial cells were exposed to dust, indicating that adhesion is not due to any direct effect of HDE on neutrophils (Fig 3B).

### 2.4 Alcohol decreases in vivo HDE-mediated ICAM-1 expression in murine lung tissue

**Figure 4:**
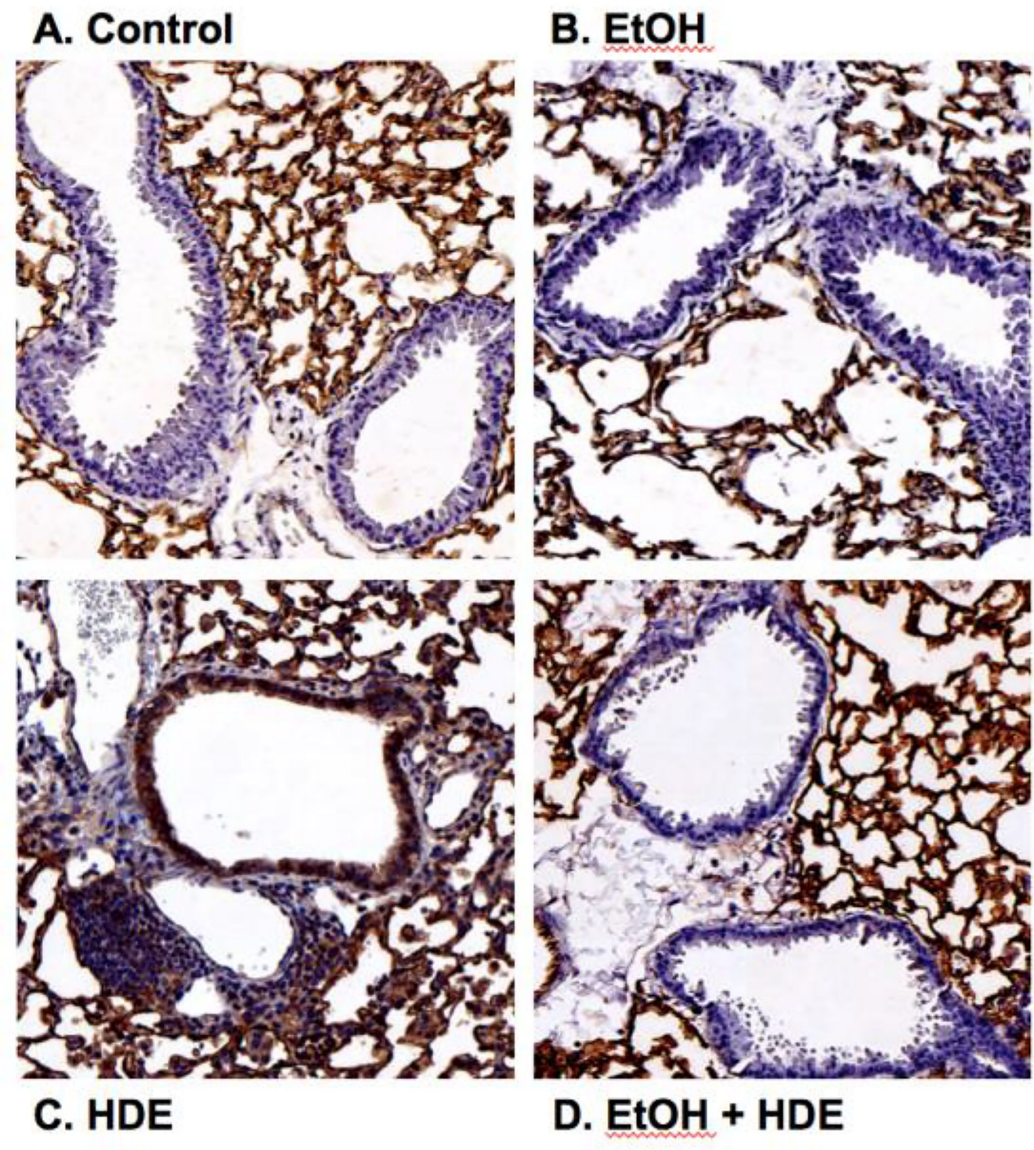
Alcohol blocks ICAM-1 localization in airway epithelium of mice instilled with HDE Mice were fed either water (control) or 20% ethanol (EtOH) for 8 wk with or without intranasal instillation of HDE. Saline inhalation in either control (A) or EtOH-fed mice (B) exhibit minimal airway epithelial ICAM-1 staining in luminal cells. Mice inhaling HDE (C) show prominent ICAM-1 staining (brown) on apical regions of the luminal airway epithelium and extensive mononuclear cell aggregates (purple). Ethanol pretreatment (D) blocks the HDE-stimulated upregulation of localized ICAM-1. Images are representative of sections from at least three mice per treatment group.

Because HDE-stimulated expression of ICAM-1 in isolated epithelial cells is attenuated by alcohol in vitro, we examined the effect of alcohol on ICAM-1 in an in vivo mouse model of inhaled HDE. Mice were allowed to drink water or 20% ethanol ad libitum for 8 wk, followed by a daily regimen of intranasal inhalation (50 µL) of sterile 12.5% HDE or sterile saline during the last 2 wk of the experiment. Similar to previously published data [17], total lung lavage cells increased with HDE treatment, but a significant reduction in neutrophils was observed in ethanol-fed mice instilled with HDE (data not shown).Fixed and paraffin-embedded lungs from exposed mice were immunostained for ICAM-1. While lung parenchymal tissue stained uniformly for ICAM-1 in all conditions, only the airway epithelium from mice treated with HDE stained positively for ICAM-1 (Fig 4). Much of this dust-enhanced ICAM-1 expression was localized to the apical surfaces of the epithelial cells lining the lumen of the airways. The airway epithelium of saline-instilled control- and ethanol-fed mice did not stain for ICAM-1. Importantly, the ethanol-fed mice instilled with HDE demonstrated a profound reduction in ICAM-1 airway epithelial staining in comparison to those mice inhaling HDE and drinking water. These findings indicate that ethanol consumption in vivo can decrease the upregulation of dust-induced ICAM-1 expression.

TACE activation is required for HDE-mediated ICAM-1 expression, neutrophil adhesion and TNFα release in bronchial epithelial cells

**Figure 5:**
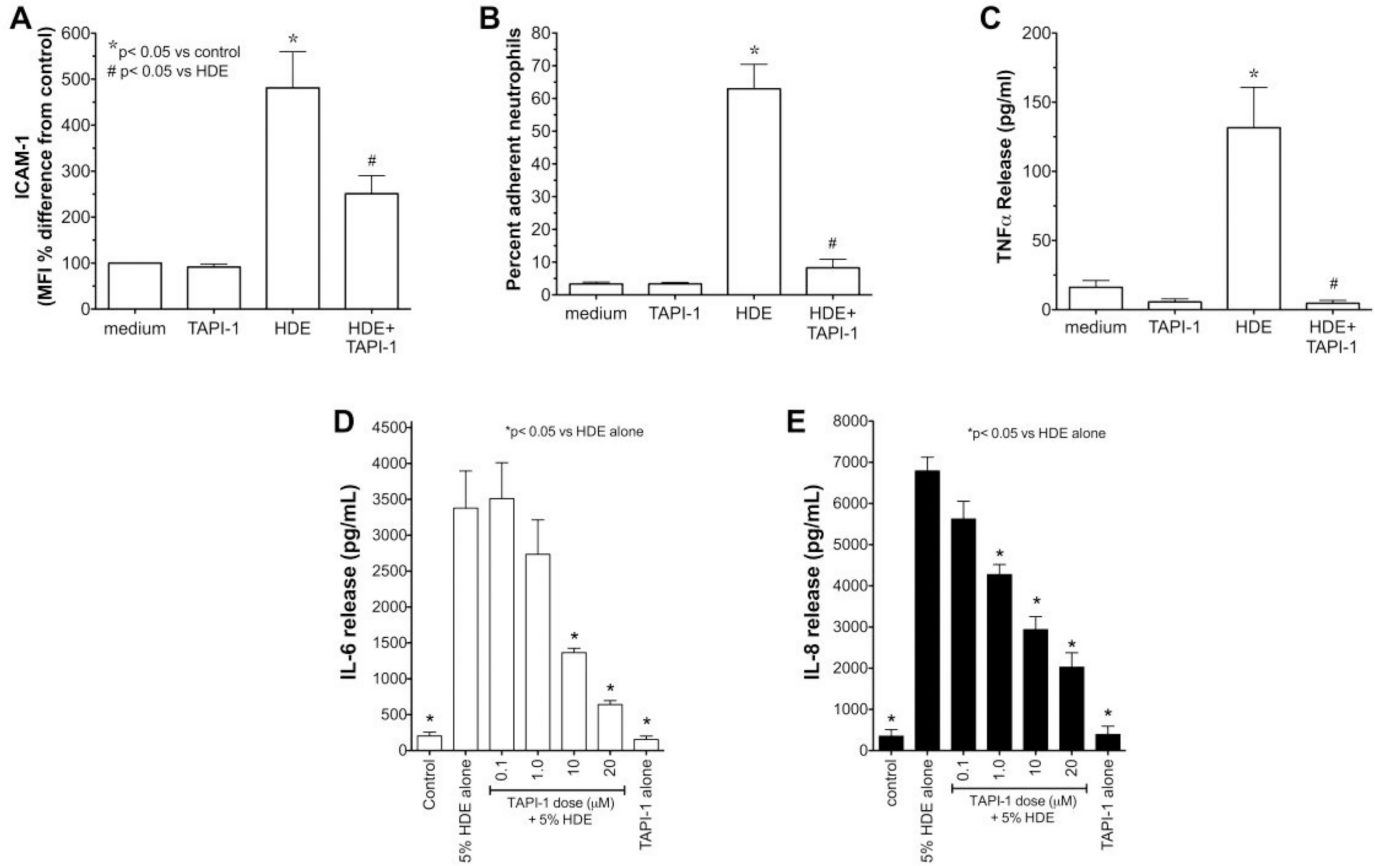
A–C: *Pretreatment with TACE inhibitor (TAPI-1) attenuates HDE-mediated ICAM-1 expression (A), PMN adhesion (B), and TNFα release (C) in human bronchial epithelial cells.* BEAS-2B cells were treated with control medium, or 5% HDE with or without 20 µM TAPI-1 for 24 hr and FACS for ICAM-1, PMN adhesion assays, and TNFα ELISA on supernates were performed. Bar graphs represent mean values with standard error bars (N=minimum of 3 independent experiments), *p<0.05 vs. control medium, #p<0.05 vs. HDE. D–E: *Pretreatment with TAPI-1 dose-dependently decreases HDE-induced cytokine release from BEAS-2B.* BEAS-2B were treated with culture medium alone, 5% HDE, 20 µM TACE inhibitor TAPI-1 alone, or were pretreated with various doses of TAPI-1 for 1 hr before exposure to 5% HDE in the presence of TAPI-1 for 24 hr. Conditioned supernatant media were assayed for IL-6 (panel D) and IL-8 (panel E) by ELISA. Bar graphs represent the means with standard error bars of 3 independent experiments (* p<0.05 vs. HDE alone).

We previously showed that alcohol can block ADAM-17 mediated release of TNFα in HDE-stimulated epithelium [16]. Thus, we investigated the role of TACE and TNFα in the down regulation of HDE-induced ICAM-1 epithelial expression and neutrophil adhesion. Confluent monolayers of BEAS-2B cells were pretreated with 20 µM TAPI-1, a TNFα converting enzyme inhibitor, for 1 hr before exposure to 5% HDE for 24 hr and ICAM-1 surface expression was measured by FACS. While 5% HDE stimulated a significant (p<0.05) increase in ICAM-1 expression, pretreatment with TAPI-1 significantly (p<0.05) decreased HDE-stimulated ICAM-1 (Fig 5A). By itself, TAPI-1 had no effect on baseline ICAM-1 MFI. Next, we examined the effect of TAPI-1on HDE-mediated neutrophil adhesion. Pretreatment (1 hr) of BEAS-2B with 20 µM TAPI-1 before exposure to 5% HDE for 24 hr significantly (p<0.05) decreased HDE-induced increases in neutrophil adhesion (Fig 5B).Again, pretreatment with TAPI-1 alone had no effect on neutrophil adherence to epithelial cells as compared to medium control conditions. Functionally, TAPI-1 pretreatment as above blocked all HDE-stimulated release of TNFα as quantified by ELISA (Fig 5C). Finally, the subsequent HDE-stimulated IL-6 and IL-8 release that occurs downstream of TNFα release (as reported in [14]) is dose-dependently inhibited by TAPI-1 pretreatment (Fig 5D–E). Collectively, these data establish the effectiveness of TACE blockade in HDE-stimulated proinflammatory cytokine release and implicate TACE-dependent TNFα release in the dust-stimulated increase in ICAM-1-mediated neutrophil adhesion.

### 2.5 Alcohol inhibits TACE activity in primary HBEC

**Figure 6:**
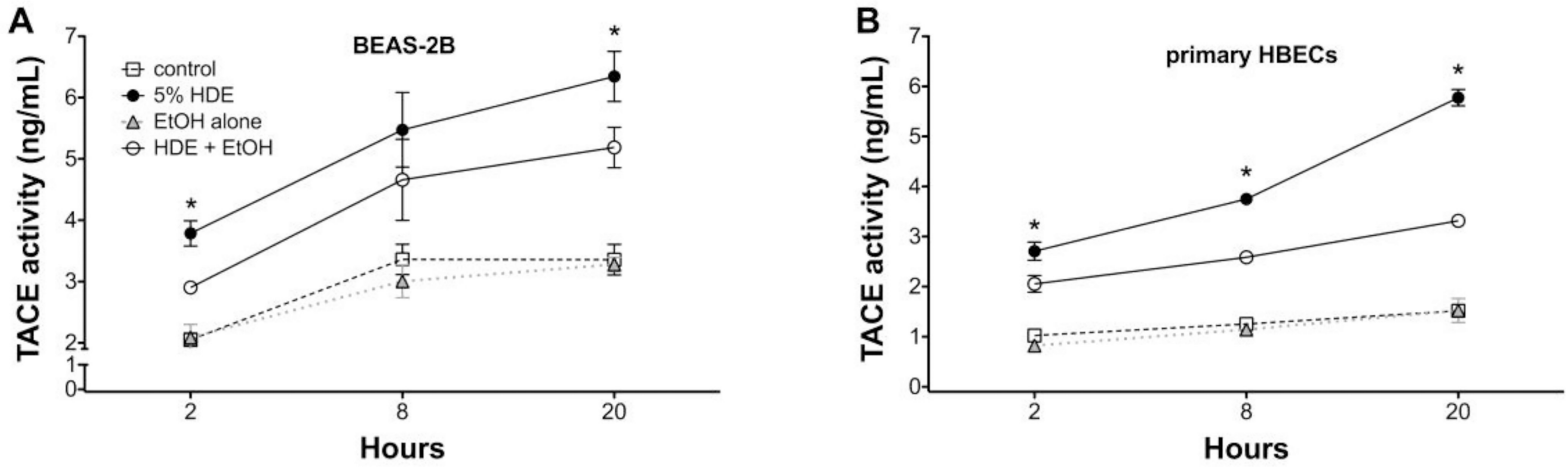
Alcohol pre-treatment inhibits HDE-induced TACE activity in BEAS-2B and primary human bronchial epithelial cells Lysates from BEAS-2B (panel A) primary human bronchial epithelial cells (HBECs; panel B) pretreated with or without 100 mM ethanol for 1 hr before exposure to 5% HDE or control medium for an additional 2, 8 or 20 hr were assayed for TACE activity. HDE stimulated TACE from 2-20 hr, but alcohol pretreatment blunted HDE-induced TACE activity at all time points. Data shown represent the means (+/− SEM) of 3 separate experiments (* p<0.05 vs. HDE+EtOH).

**Figure 7:**
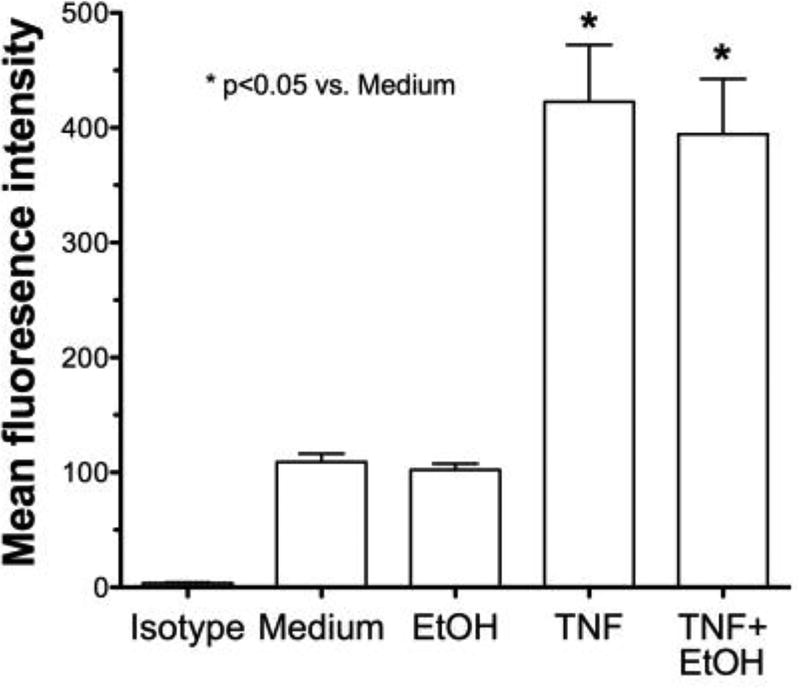
Alcohol does not directly affect TNFα-induced ICAM-1 expression BEAS-2B were treated with either TNFα (0.1 ng/ml) or TNFα incubated with 100 mM ethanol for 1 hr before epithelial cell treatment. FACS for ICAM-1 revealed a significant increase in ICAM-1 expression even when cells were treated with TNFα + ethanol. Bar graphs represent the mean with standard error bars of 3 separate experiments (* p<0.05 vs. control medium).

To determine if alcohol is decreasing TNFα through TACE inhibition and causing the observed decrease in ICAM-1 and neutrophil adhesion, we examined the direct affect of alcohol on TACE activity in airway epithelial cells. Both BEAS-2B (Fig 6A) and HBECs (Fig 6B) were pretreated with ethanol for 1 hr before exposure to 5% HDE or control media for an additional 2, 8 or 20 hr. At all time points examined, there was a significant (p<0.05) increase in TACE activity with HDE treatment. However, cells pretreated with 100 mM ethanol followed by HDE exposure demonstrated a considerable reduction in TACE activity at all time points. TACE activity in cells treated with alcohol alone was no different from the control medium. Furthermore, to control for any non-specific direct effect of alcohol on TNFα itself, TNFα (0.1 ng/ml) was mixed with 100 mM ethanol and incubated for 1 hr before the combination was used to treat epithelial cells. FACS for ICAM-1 revealed that there was a significant increase in ICAM-1 expression even when cells were treated with the TNFα + ethanol mixture (Fig 7). This increased ICAM-1 expression was similar to the response seen when treating the cells with TNFα only and indicates that the alcohol does not directly alter TNFα, itself. These data demonstrate that alcohol directly affects TACE activity, leading to decreases in the release of endogenous TNFα, and results in reduced ICAM-1-mediated neutrophil adhesion.

## 3. Discussion

Alcohol is increasingly recognized as an important immunomodulator of lung responses because it can impair lung immunity by affecting cytokines instrumental in inflammatory responses. This lung immunity impairment results in clinical problems including a significantly enhanced susceptibility to pneumonia [25]. Likewise, our results demonstrate that ethanol modifies the normative lung inflammatory response to hog barn dust extract in human bronchial epithelial cells. We and others have established in previous studies that inhaled swine dusts induce lung inflammation associated with the elevation of TNFα, IL-6, IL-8, ICAM-1, and neutrophil adhesion [26–29]. In the present study, we have shown that alcohol decreases swine barn dust-mediated ICAM-1 expression, neutrophil adhesion and stimulated cytokine release of TNFα, IL-6, and IL-8. We have also demonstrated that alcohol decreases HDE-mediated ICAM-1 expression *in vivo* using a murine model of alcohol feeding and inhaled hog CAFO dust exposure. Collectively, these studies add to the growing body of literature demonstrating an important immunomodulatory role for alcohol in impairing lung immunity.

Studies have shown that alcohol compromises the immune system [30] and increases the risk of lung infections and bacterial pneumonia [31]. Environmental exposures of any kind, including agricultural organic dusts, subject the lungs to inhalation-based injury. The first line of defense other than exhalation or cough is the physical barrier represented by mucociliary transport. Alcohol-induced ciliary dysfunction (AICD) compromises the innate ability to effectively clear dust particles and their associated toxins from the lungs in a typical timely manner [32]. Furthermore, alcohol depletes lung antioxidants, resulting in the loss of effective barrier function and leading to enhanced lung edema [33, 34]. In addition, alcohol alters normative immune effector cell function causing alterations in cytokine production and responses that impair adaptive immunity [35, 36]. Collectively, this concept has been referred to as the “Alcoholic Lung” [37] and should be considered within the context of all inhalation injury including cigarette smoking [38], burn injury [39], and occupational dust exposure.

We identified the highest relevant dose of alcohol that would alter dust-mediated effects without producing cytotoxicity (Fig 1). The data obtained in our experimental model utilizes a concentration of alcohol relevant to public health. Humans tolerate ethanol in extremely high concentrations with low levels of toxicity. Normal human metabolism and microflora content results in daily endogenous production of µM blood alcohol concentration (BAC) [40]. Oral alcohol ingestion rapidly elevates BAC so significantly that 1–2 standard drinks can result in 8–10 mM (40 mg/dL) levels. Problematic BAC ranges from legal intoxication levels (22 mM; 100 mg/dL) to pathophysiologic concentrations requiring medical attention (100–200 mM; 400–900 mg/dL); a record case report documented a BAC of 328 mM (1510 mg/dL) [41]. To compound matters, localized concentrations of alcohol in the airways of the lung can be significantly elevated beyond that of the BAC due to the condensation or “rain effect” of exhaled alcohol vapor [32]. Therefore, co-exposure studies that utilize 100 mM concentrations of ethanol are justified within the context of lung exposures to inhaled dust in individuals with alcohol use disorders.

Previously we have reported that HDE increases adhesion molecule ICAM-1 surface expression on bronchial epithelial cells [19]. ICAM-1 is an adhesion molecule that facilitates the interaction of neutrophils with bronchial epithelial cells, leading to enhanced neutrophil functioning [42, 43]. Thus, the upregulation of ICAM-1 on the surface of bronchial epithelial cells following an exogenous inflammatory insult like organic dusts is important to the productive immune response. Reduced ICAM-1 responsiveness could alter lung inflammatory responses and negatively impact lung immunity. We have shown that dust-mediated upregulation of ICAM-1 is dependent upon the TNFα-stimulated activation of PKCε [14]. The epithelium itself can be an autocrine source of this TNFα in response to the swine barn dust in that various components of the dust, such as peptidoglycan and protease activity, lead to the TLR2/MyD88-dependent activation of PKCα to generate sufficient TACE activity for TNFα release [14, 44, 45]. Recently, we have found that a cAMP-dependent pathway mediated by PKA is capable of inhibiting dust-stimulated TACE activation [15]. This effect is rapid and not the result of TACE down-regulation. This is particularly important in the context of alcohol exposure as an ethanol-sensitive adenylyl cyclase in airway epithelium [46] can also lead to the inhibition of dust-stimulated TACE activity [16]. Such alcohol-mediated alterations in response to swine barn dust may explain the significant lack of inflammatory cell aggregates in the lungs of mice fed alcohol prior to nasal inhalation of swine barn dust [17].

The precise and complete mechanism of alcohol-modulation of the TNFα response to swine barn dust inhalation is not known. It has been established in multiple studies that HDE increases release of TNFα and stimulates ICAM-1 expression in bronchial epithelial cells [17]. We hypothesized that the swine dust-induced increase in ICAM-1 occurs through a TNFα pathway and it is this pathway that is affected by alcohol, resulting in the down regulation of ICAM-1 with alcohol exposure. We also looked at the effects of alcohol pretreatment on purified TNFα-induced ICAM-1 expression, but observed no inhibition or decrease. This indicates that alcohol does not directly block TNFα downstream signaling, but rather must interfere with an upstream precursor to TNFα release. By using a direct inhibitor of the sheddase responsible for TNFα cleavage (activation) and release (TAPI-1), we were able to demonstrate the requirement for dust-stimulated TACE activation in the upregulated expression of ICAM-1 on the apical surface of the airway epithelium. This supports our previous finding that agents that are capable of elevating cAMP levels and activating PKA are capable of inhibiting TACE [15]. In addition to our previous work demonstrating that alcohol can rapidly stimulate cAMP and activate PKA in airway epithelium [47], others have previously established that ethanol exposure can lead to TACE inhibition in monocytes [48] and an alveolar epithelial cell line, A549 [49]. It remains unknown at this time whether PKA interacts directly or indirectly with TACE via cAMP-mediated phosphorylation and how such an interaction blocks the sheddase action of TACE on facilitating TNFα release.

In conclusion, the findings presented here support our previous observations that the combination of alcohol consumption and organic dust inhalation results in an enhanced injury, not a protection against inflammation. Alcohol suppression of ICAM-1 expression adds to the mechanistic basis for the dysfunctional lung inflammatory response, weight loss, and mortality observed in alcohol-fed mice exposed to swine barn dust [17]. In addition to TNFα, the systemic effect of alcohol on multiple cytokine responses in the normative repair response to injury likely complicates the role of ethanol-mediated TACE inhibition. Inflammatory responses to injury are not necessarily pathologic and indeed represent a continuum toward eventual repair and homeostasis. This alcohol-mediated alteration to the normative lung response to dust injury does not translate into the prevention or lessening of that injury. Strategies to manage chronic lung injury from the repeated pro-inflammatory response to inhaled organic dusts might involve the manipulation of the cAMP pathway through pharmacologic intervention. In contrast, complications of initiating a proper and protective inflammatory response to dust exposure may be elicited by heavy alcohol exposure. Thus, alcohol use status may be a valuable consideration in the management of agricultural occupational injury.

## 4. Materials and Methods

### 4.1 Cell preparation

BEAS-2B cells (American Type Culture Collection, Manassas, VA), an SV40-transformed human bronchial epithelial cell line [18], were grown on type I collagen-coated dishes in a serum-free LHC9-RPMI 1640 (1:1) mixture (containing inorganic salts, bovine pituitary extract, trace elements, epidermal growth factor, insulin, triiodothyronine, epinephrine, calcium, retinoic acid, hydrocortisone, human transferrin, penicillin and streptomycin) and maintained at 37°C in 5% CO_2_, as previously described [13]. Primary human bronchial epithelial cells (HBEC) were isolated from human lung tissue obtained from the International Institute for the Advancement of Medicine (IIAM; Edison, NJ), a repository for organs and tissue donated for transplantation, but determined to be unsuitable for various reasons. Written informed consent for use of these tissues was obtained by IIAM from the donor or next of kin in compliance with all applicable state and federal laws, and experiments were conducted under UNMC IRB approval. Lung specimens were infused with histidine-tryptophan-ketoglutarate (HTK) medium and shipped on ice to our facility via courier. Isolation of bronchial epithelial cells was begun immediately upon arrival.

Shipping medium was decanted and the mainstem, lobar, and segmental bronchi were exposed and dissected into 0.5–2 cm segments. Bronchial segments were then submerged in a mucolytic buffer (0.5 µg/mL dithiothreitol, 10µg/mL DNase I, in Dulbecco's modified Eagle's medium (DMEM) containing fungicide and antibiotics) with agitation for 20 min. The tissue was rinsed twice with DMEM before incubation in proteolytic digestion buffer (0.1% trypsin/EDTA, 10µg/mL DNase I in DMEM) for 48 hr with agitation at 4°C. Proteases were then neutralized with 10% fetal calf serum and bronchial segments sliced lengthwise and the luminal surface scraped with a sterile scalpel. Disaggregated cells were collected and strained through a 40 µm pore size cell strainer, washed twice in cold DMEM and counted. Single-cell suspensions were plated on type I collagen-coated 100 mm tissue culture dishes and maintained in growth factor-supplemented, serum-free BEGM (bronchial epithelial growth medium, Lonza, Walkersville, MD) in a 5% CO_2_-enriched and humidified atmosphere at 37°C. Cell cultures were monitored and fed every second day. Cells derived from these initial cultures were either cryopreserved under liquid nitrogen, or passaged up to five times for use in these experiments.

### 4.2 Preparation of HDE

Dust samples were collected from Nebraska hog confinement facilities consisting of approximately 1000 hogs and were used to prepare extract as previously described [19]. Briefly, 1 g of dust was suspended in 10 ml of Hanks’ Balanced Salt Solution (HBSS) and mixed on a magnetic stir plate for 1 hr at room temperature. The mixture was centrifuged at 400 g for 20 min at 4°C, the supernate collected and re-centrifuged at 400 g for an additional 20 min at 4°C. The supernate was filter-sterilized (0.2 µm) and frozen in aliquots at −20°C. The aqueous dust extract was diluted to a concentration of 5% in growth medium for these experiments. Extracts prepared in this fashion contain only ultrafine particulates that pass through the 0.2 µm filter.

### 4.3 Flow cytometry analysis for epithelial cells

BEAS-2B cells were plated on six-well plates and grown to confluency. Cells were first exposed to ethanol (100 mM) or the TACE inhibitor, TAPI-1 (20 µM), for 1hr prior to stimulation with or without 5% HDE for an additional 24 hr. Cells were harvested with 0.05% trypsin-EDTA at 37°C and the trypsin neutralized with the addition of 0.2 ml of 0.2% soybean trypsin inhibitor. Cells were centrifuged and washed in phosphate buffered saline (PBS; pH 7.4), then centrifuged again and fixed with 2 ml of a 1% paraformaldehyde solution (PFA) followed by two washings with 2 ml PBS. The cells were stained with PE-conjugated anti-human CD54 antibody or with anisotype control PE-conjugated anti-human IgG antibody and incubated for 85 min on ice. The cells were centrifuged and washed with PBS, followed by the addition of 1 ml 1% PFA. The cells were then analyzed using a FACSCaliber flow cytometer and the results analyzed using BD CellQuest software (Becton Dickinson, San Jose, CA). The mean fluorescence intensity (MFI) was reported for 10,000 events for each condition. Results are expressed as the MFI (+/− SEM) of three or more parallel experiments.

### 4.4 Neutrophil preparation

Human blood neutrophils (PMN) were obtained from normal volunteers on a protocol approved by the Human Studies Subcommittee of the Research and Development committee of the Omaha Veterans Affairs Medical Center. Peripheral venous blood (20 mL) was collected in an acid-citrate/dextrose (ACD) preservative-treated tube and immediately mixed with a 6% dextran solution to allow for RBC sedimentation. After 45 min, the leukocyte-enriched supernate was removed and subjected to RBC lysis in an ammonium chloride buffer. Mononuclear cells and lymphocytes were removed from the leukocyte component by fractionation over a Ficoll gradient (Histopaque 1077, Sigma, St Louis, MO). The washed pellet (30 ×10^6^ PMN) was stained with the vital fluorescent chromogen calcein AM (calcein acetolymethyl ester, EMD, San Diego, CA) at 10 µg/mL/1×10^6^ cells and incubated on ice for 30 min in the dark. The stained neutrophils were washed three times with PBS and suspended at a concentration of 1 × 10^6^cells/mL in RPMI basal medium before beginning co-culture with previously treated BEAS-2B monolayers.

### 4.5 Neutrophil adhesion assay

BEAS-2B cells were grown to confluency on collagen-coated 24-well plates. Cells were exposed to ethanol (100 mM) or TAPI-1 (20 µM) for 1hr before being challenged with or without 5% HDE for an additional 24 hr, as described above for cytometry experiments. Co-culture consisted of 5 × 10^5^ neutrophils per well of a confluent 24-well cluster plate, resulting in an effective PMN to epithelial cell ratio of 2.25:1. At no time were PMN exposed to dust extract. After 30 min at 37°C, non-adherent neutrophils were thoroughly removed from the plates by serial PBS washes. The attached neutrophils and epithelial cell layers were removed with a 0.05% trypsin solution. The cell pellets were then transferred to individual wells of a black 96-well fluorescence microtiter plate along with a standard curve consisting of calcein AM stained neutrophils. Six replicates for each experimental condition were measured. Samples were read in a Fluorolite fluorescence spectrophotometer (Dynatech, Chantilly, VA), using excitation/emission wavelengths of 490nm/530nm respectively. Data interpolated from the standard curve as adherent cells per well was then converted to percent adherence based on the 5 × 10^5^ cells available for adherence in each well.

### 4.6 Protein kinase activity assay

Direct measurement of catalytic activities of the cAMP-dependent protein kinase (PKA) and protein kinase C-epsilon (PKCε) were determined as previously described [15, 20].

### 4.7 Cytokine release assay

Release of IL-6 and IL-8 was assayed by ELISA as previously reported [13]. TNFα levels were quantified in duplicate for each experiment using a sandwich ELISA as follows: 96-well flat-bottomed polystyrene microtiter plates were coated with 100 µl/well of mouse anti-human TNFα (R&D Systems, Minneapolis, MN) diluted 1:250 in Voller’s buffer overnight at 4°C. The plate was washed three times with PBS-Tween, and undiluted culture supernates or standard human recombinant TNFα (R&D) were applied to the plates and incubated at room temperature for 2 hr. The plate was washed again with PBS-Tween, and biotinylated goat anti-human TNFα antibody diluted 1:250 in PBS-Tween was applied to the plate and incubated at room temperature for 2 hr. After three washes, streptavidin-HRP diluted 1:200 in PBS-Tween was added for 20 min. The plate was again washedand incubated with a TMB/peroxidase substrate (R&D) for 30 min. The reaction was terminated with 27.5 µl/well of 8M sulfuric acid and plate was read at 450 nm in an automated ELISA reader (MRX Revelation, Dynex Technologies, Chantilly, VA).

### 4.8 Animal model and exposure

All experimental procedures were reviewed and approved by the Institutional Animal Care and Use Committee of the Veterans Affairs Research Service, Nebraska-Western Iowa Health Care System and the University of Nebraska Medical Center (UNMC).

Male C57BL/6 mice (age 8 wk), obtained from Jackson Labs (Bar Harbor, ME), were randomly assigned to a control or alcohol treatment group. The alcohol group received *ad libitum*ethanol (20%) in drinking water for 8 wk as established by the Meadows-Cook model [21, 22]. This alcohol feeding model consistently produces a blood alcohol concentration (BAC) range of 10–80 mM in mice and approximates BAC commonly observed in human alcohol use disorders. Each group was treated with daily intranasal instillation of 12.5% HDE or saline during the last 2 wk as previously described [23]. This concentration of dust extract instillation models creates a consistent lung pathology of pronounced lymphoid aggregates similar to inducible bronchus-associated lymphoid tissue (iBALT) that facilitates unambiguous scoring. The mice were sacrificed, lungs removed *en bloc*, inflated and fixed in formalin, embedded in paraffin and thin sections (4–5 mm) were prepared by the UNMC histology core laboratory.

### 4.9 Immunohistochemistry

Formalin-fixed, paraffin-embedded sections of tissue were deparaffinized and rehydrated in graded alcohol washes. Antigen unmasking was performed using the heat-induced epitope retrieval method with Diva Decloaker (Biocare Medical, Concord, CA). Endogenous peroxidase activity was quenched with 3% hydrogen peroxide and slides blocked in 10% rabbit serum before application of primary antibody. Slides were incubated with purified rat anti-mouse CD54 (Biolegend, SanDiego, CA) diluted 1:200 overnight at 4°C. The slides were washed and incubated with biotinylated goat anti-rat IgG (Biolegend, 1:500) for 1hr at room temperature. The slides were washed and the avidin-biotin-immunoperoxidase technique (Vectastain Elite ABC RTU kit, Vector Labs, Burlingame, CA) used to detect primary binding. The slides were developed with Chromogen substrate (ImmPACT DAB, Vector) and counterstained with hematoxylin. Slides were rehydrated in graded alcohol, cleared with xylene, coverslipped and analyzed microscopically.

### 4.10 TACE activity assay

TNF converting enzyme (TACE; ADAM-17) levels were measured in primary epithelial cell lysates using a microplate-format colorimetric assay kit (human TACE EIA Duoset, R&D Systems, Minneapolis, MN). Epithelial cells grown to 90% confluence on 12-well plates were pretreated with 100 mM ethanol or with the TACE inhibitor, TAPI-1 (20 M), for 1 hr before being challenged with or without 5% HDE for an additional 2, 8, or 20 hr. Cells were gently removed from the plate using warm 1 mM EDTA, washed, and lysed in cold MgCl_2_/Tris/EGTA lysing buffer containing protease inhibitors (protease inhibitor cocktail, Sigma, St. Louis, MO), and the membrane-free lysates assayed for TACE activity following the kit manufacturer’s instructions. TACE activity in each sample was determined by interpolating absorption at 450 nm from a standard curve of recombinant human TACE and is expressed as ng/mL.

### 4.11 Cell viability assay

Supernatant media (50 µL) from cultured cell monolayers under treatment conditions or media alone were assayed for viability using a TOX-7 kit (Sigma) to measure lactate dehydrogenase (LDH) release, as permanufacturer instructions. As a positive control, confluent cells were lysed (0.1% Triton-X 100) and total LDH release measured.

### 4.12 Materials

All materials not specifically indicated were obtained from Sigma-Aldrich (St. Louis, MO).

### 4.13 Statistical analysis

Data are represented as means +/− standard errors (SEM) for values pooled from 3 or more parallel experiments. All pair-wise comparisons were examined using a one-way ANOVA with Tukey’s post hoc analysis (GraphPad Prism software, San Diego, CA). Statistical significance was accepted when *P*-values were ≤ 0.05.
